# Phase 1/2a Trial of *Plasmodium vivax* Malaria Vaccine Candidate VMP001/AS01_B_ in Malaria-Naive Adults: Safety, Immunogenicity, and Efficacy

**DOI:** 10.1371/journal.pntd.0004423

**Published:** 2016-02-26

**Authors:** Jason W. Bennett, Anjali Yadava, Donna Tosh, Jetsumon Sattabongkot, Jack Komisar, Lisa A. Ware, William F. McCarthy, Jessica J. Cowden, Jason Regules, Michele D. Spring, Kristopher Paolino, Joshua D. Hartzell, James F. Cummings, Thomas L. Richie, Joanne Lumsden, Edwin Kamau, Jittawadee Murphy, Cynthia Lee, Falgunee Parekh, Ashley Birkett, Joe Cohen, W. Ripley Ballou, Mark E. Polhemus, Yannick F. Vanloubbeeck, Johan Vekemans, Christian F. Ockenhouse

**Affiliations:** 1 Malaria Vaccine Brach, Walter Reed Army Institute of Research, Silver Spring, Maryland, United States of America; 2 Armed Forces Research Institute of Medical Sciences, Bangkok, Thailand; 3 U.S. Army Medical Materiel Development Activity, Frederick, Maryland, United States of America; 4 Walter Reed National Military Medical Center, Bethesda, Maryland, United States of America; 5 Naval Medical Research Center, Silver Spring, Maryland, United States of America; 6 PATH-MVI, Washington, D.C., United States of America; 7 GSK Vaccines, Rixensart, Belgium; Johns Hopkins Bloomberg School of Public Health, UNITED STATES

## Abstract

**Background:**

A vaccine to prevent infection and disease caused by *Plasmodium vivax* is needed both to reduce the morbidity caused by this parasite and as a key component in efforts to eradicate malaria worldwide. Vivax malaria protein 1 (VMP001), a novel chimeric protein that incorporates the amino- and carboxy- terminal regions of the circumsporozoite protein (CSP) and a truncated repeat region that contains repeat sequences from both the VK210 (type 1) and the VK247 (type 2) parasites, was developed as a vaccine candidate for global use.

**Methods:**

We conducted a first-in-human Phase 1 dose escalation vaccine study with controlled human malaria infection (CHMI) of VMP001 formulated in the GSK Adjuvant System AS01_B_. A total of 30 volunteers divided into 3 groups (10 per group) were given 3 intramuscular injections of 15μg, 30μg, or 60μg respectively of VMP001, all formulated in 500μL of AS01_B_ at each immunization. All vaccinated volunteers participated in a *P*. *vivax* CHMI 14 days following the third immunization. Six non-vaccinated subjects served as infectivity controls.

**Results:**

The vaccine was shown to be well tolerated and immunogenic. All volunteers generated robust humoral and cellular immune responses to the vaccine antigen. Vaccination did not induce sterile protection; however, a small but significant delay in time to parasitemia was seen in 59% of vaccinated subjects compared to the control group. An association was identified between levels of anti-type 1 repeat antibodies and prepatent period.

**Significance:**

This trial was the first to assess the efficacy of a *P*. *vivax* CSP vaccine candidate by CHMI. The association of type 1 repeat-specific antibody responses with delay in the prepatency period suggests that augmenting the immune responses to this domain may improve strain-specific vaccine efficacy. The availability of a *P*. *vivax* CHMI model will accelerate the process of *P*. *vivax* vaccine development, allowing better selection of candidate vaccines for advancement to field trials.

## Author Summary

*Plasmodium vivax* malaria has several unique features. Two of the main features are the inability to culture this parasite in vitro and its propensity to form dormant stages within the liver, which can only be treated with a single class of drugs that are contraindicated for a proportion of the population. Therefore, vaccines will play an important role in preventing this geographically widespread malaria species. In this clinical trial, we tested increasing amounts of the vaccine candidate VMP001/AS01_B_ for safety and immunogenicity. In order to test if the vaccine can afford protection, we challenged the volunteers via the bite of infected mosquitoes, the first time such a human infection model has been used to evaluate vaccine efficacy for *P*. *vivax* malaria. While the vaccine did not protect any of the vaccinated subjects, this study resulted in some important findings, including the observation that a significant proportion of the subjects displayed a trend towards a delay in infection in individuals that correlated with antibodies to the repeat region of the vaccine antigen.

## Introduction

Malaria is a devastating parasitic disease transmitted through the bite of infected *Anopheles* mosquitoes. Outside sub-Saharan Africa, *Plasmodium vivax* is the most prevalent of all human malarias with approximately 2.48 billion people at risk [1] and an estimated 16 million cases in 2013 (WHO World Malaria Report, 2014). Unlike *Plasmodium falciparum*, *P*. *vivax* produces liver stages (hypnozoites) that, initially dormant, can reactivate several weeks to months after the primary infection causing symptomatic disease [2,3]. This propensity to relapse stands as a significant barrier to efforts to eradicate this species of malaria [3]. Additionally, *P*. *vivax* is increasingly reported as the causative agent of symptoms associated with severe malaria as well as chloroquine resistance [4–7]. A vaccine to prevent infection and disease caused by *P*. *vivax* is urgently needed to reduce morbidity of the disease and accelerate elimination of this parasite.

The circumsporozoite protein (CSP) is the most abundant sporozoite protein present on the sporozoites of all *Plasmodium* species and has been shown to have great potential as a vaccine target [8,9]. Antibodies to the repeat region of *P*. *falciparum* CSP have been shown to be associated with protection [10–12]. Unlike *P*. *falciparum*, the repeat region of *P*. *vivax* CSP exhibits sequence heterogeneity resulting in immunologically distinct populations indicating that a vaccine based on one strain may not be sufficient to protect against all circulating strains [13]. To take into account the diversity of *P*. *vivax* strains, we developed vivax malaria protein 001 (VMP001) as a candidate vaccine for *P*. *vivax* malaria. The vaccine antigen VMP001 is an *Escherichia coli* produced synthetic chimeric recombinant protein that incorporates the three major domains of CSP but is distinct from the native molecule [14,15]. This synthetic construct includes the amino (N-) and carboxy (C-) terminal parts of CSP and a truncated repeat region that contains repeat sequences from the immunologically divergent VK210 (type 1) and the VK247 (type 2) strains of parasites. The VMP001 antigen was adjuvanted with AS01_B_, a proprietary liposome-based adjuvant system from GSK Biologicals that contains the immunostimulants monophosphoryl lipid A (MPL) and QS-21, a triterpene glycoside purified from the bark of *Quillaja saponaria* [9]. This adjuvant system has been used in other malaria vaccine candidates, including RTS,S [9].

We report the results of a first in humans phase 1 clinical trial using VMP001/AS01_B_ in terms of reactogenicity, immunogenicity, and efficacy against a *P*. *vivax* sporozoite challenge in healthy, malaria-naive adults.

## Materials and Methods

### Ethics Statement

The study (ClinicalTrials.gov identifier NCT01157897), sponsored by the Office of the Surgeon General, U.S. Army, was conducted following scientific and ethical review by the WRAIR scientific review committee, WRAIR institutional review board (IRB), the USAMRMC’s Human Subjects Research and Review Board as well as the Western IRB and assigned protocol numbers WRAIR 1692, HRPO A-16037. The protocol was conducted under the U.S. Food and Drug Administration (FDA) Investigational New Drug (IND) application #14380.

### Study Subjects and Eligibility

Written informed consent was obtained from all volunteers prior to screening and enrollment. Subjects were healthy malaria naïve men and women aged 18–55 years. All subjects had normal blood levels of glucose-6-phosphate dehydrogenase (G6PD), and were either homozygous or heterozygous positive for Duffy antigen. Subjects agreed to be available for the duration of study with no plans to travel to a malaria endemic area or outside the Washington, DC area until a treatment course was completed following CHMI.

### Study Design

**Fig 1:**
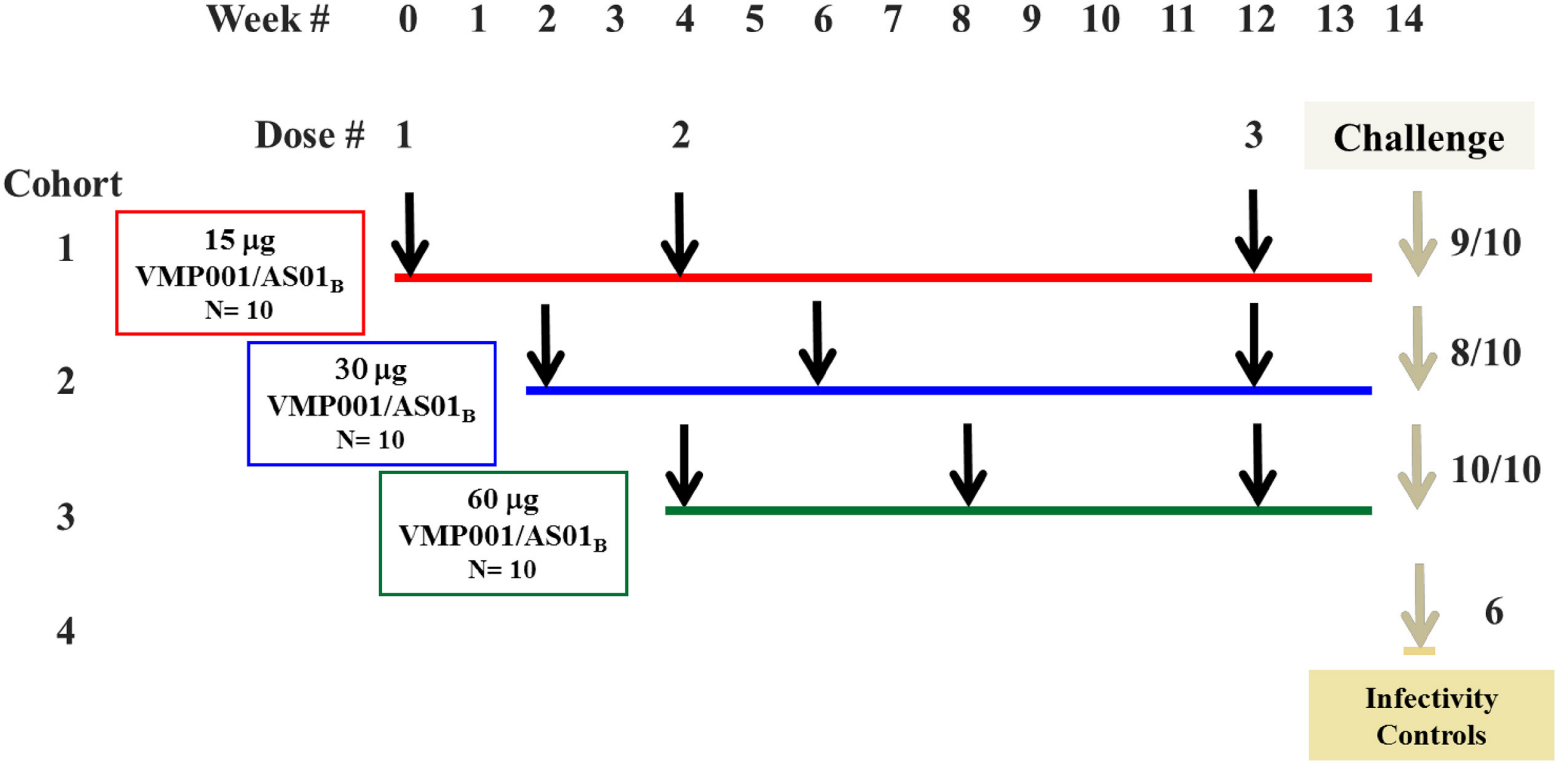
Study design. Immunization dose and schedule for the three cohorts of ten volunteers who were immunized three times with either a low (15 μg), medium (30 μg) or high (60 μg) dose of the vaccine, VMP001 formulated in AS01_B_ adjuvant (VMP001/AS01_B_). The first 2 immunizations for each cohort were staggered by two weeks in order to monitor vaccine safety. The 2^nd^ dose was delivered 4 weeks after the 1^st^ and the 3^rd^ immunization was performed 8, 6 and 4 weeks post 2^nd^ dose for cohorts 1 (red), 2 (blue) and 3 (green), respectively. Control and vaccinated subjects were challenged at WRAIR with mosquitoes infected with P. vivax parasites that originated in Thailand.

**Fig 2:**
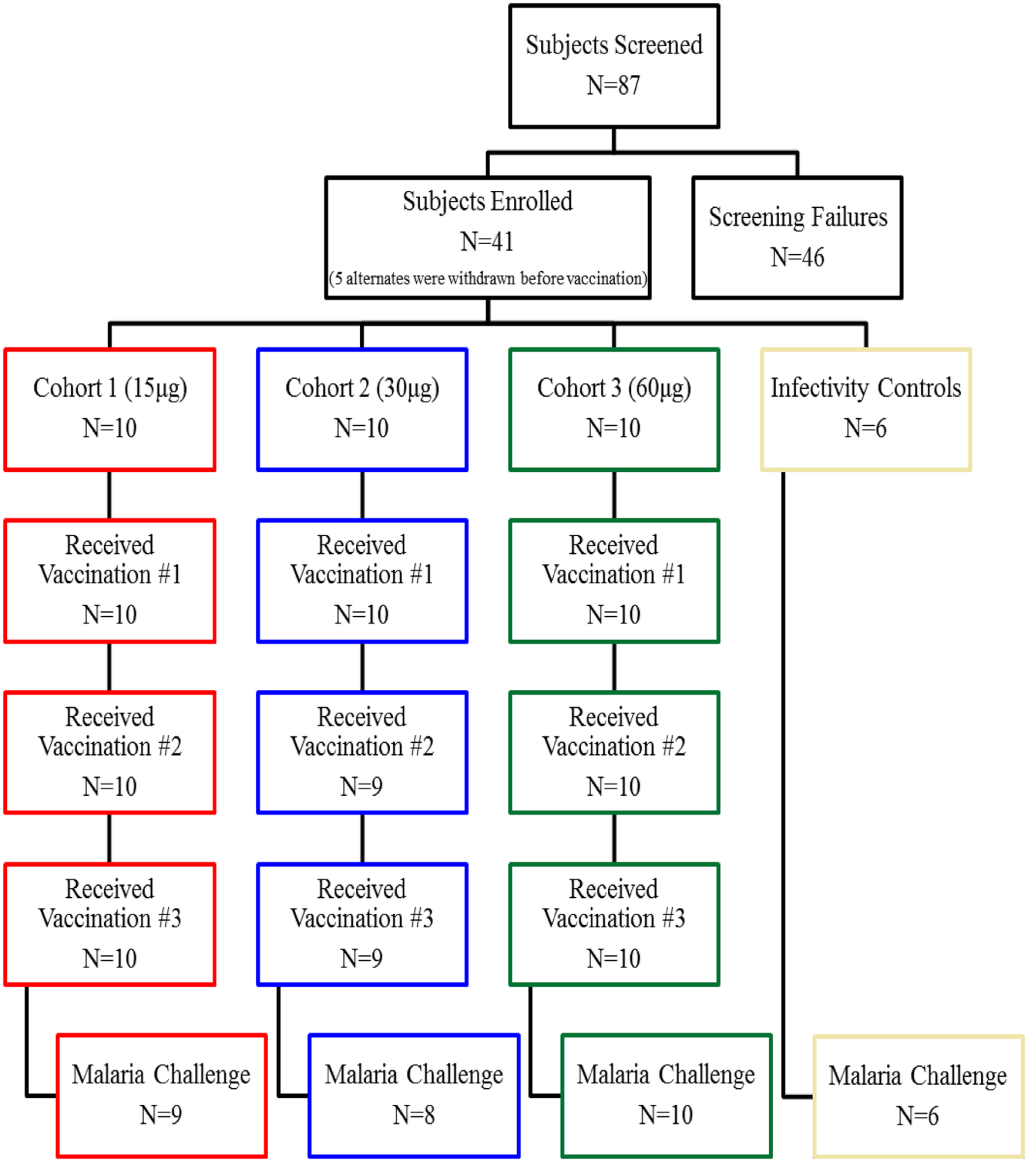
Study flow. Fig outlines the number of subjects who were screened, enrolled, and received each vaccination. A total of three volunteers, one in cohort 1 and two in cohort 2, dropped out for reasons not related to the study.

This was a phase 1, non-randomized, open label, dose-escalation study in 36 adults. Thirty volunteers, divided into 3 cohorts (10 in each group), were vaccinated with 3 doses of VMP001/AS01_B_. Cohorts 1, 2, and 3 received 15 μg, 30 μg, or 60 μg, respectively, of VMP001in 500 μL of AS01_B_ at each immunization. The first and second immunizations in each cohort were separated by 28 days, and the third dose for all cohorts was normalized such that the interval between the last immunization and day of challenge was 2 weeks (Fig 1). Controlled human malaria infection (CHMI) using *P*. *vivax* infected *Anopheles dirus* mosquitoes was performed in the volunteers from all cohorts that completed all 3 immunizations (n = 27) and a control group (n = 6) who were not administered investigational product (Figs 1 and 2).

### Vaccine

The VMP001 antigen [14,15] and the AS01_B_ adjuvant system [9] have been described previously. The VMP001 recombinant subunit protein was produced in and purified from *E*. *coli* [15] and reconstituted in 500 μl of AS01_B_. AS01_B_ is an Adjuvant System containing 50 μg 3-*O*-desacyl-4’- monophosphoryl lipid A (MPL, produced by GSK) and 50 μg *Quillaja saponaria* Molina, fraction 21 (QS-21, licensed by GSK from Antigenics Inc, a wholly owned subsidiary of Agenus Inc., a Delaware, USA corporation), in a liposomal formulation [9]. Lyophilized VMP001 was reconstituted with liquid AS01_B_ and administered in doses of 15 μg, 30 μg, or 60 μg (500 μl/dose) by slow intramuscular injection within 1 hour of reconstitution.

### Safety Assessments

Vaccine tolerability was assessed by evaluating local reactogenicity and systemic symptoms, as well as changes in biochemical and hematologic laboratory parameters. Clinical assessments of subjects were performed 30 min after each vaccination and again 1, 2, and 6 days later. Local and systemic solicited adverse events (AEs) were collected during this 7 day period. Severity of adverse reactions was classified as grade 1 (mild), grade 2 (moderate) and grade 3 (severe). Unsolicited AEs were recorded during the 28 days following vaccinations 1 and 2 and during the 14 days after vaccination 3 and reported to a safety monitoring committee (SMC). Biochemical and hematologic laboratory parameters were measured prior to administration and 6 days after each vaccination. Complete blood cell counts with white blood cell differential counts were performed in addition to serum levels of creatinine, alanine aminotransferase, and aspartate aminotransferase.

During the challenge phase, solicited AEs were recorded beginning on the day of CHMI. Clinical assessments continued on days 1 and 3 post-CHMI, and then daily beginning on day 5 until the subject had 3 consecutive negative daily blood smears following the initiation of treatment for malaria infection. All unsolicited events were documented for 28 days beginning on the day of CMHI. Safety laboratories were measured on the day of CHMI, at the time of initiating anti-malaria therapy, and on days 28 and 42 post-CMHI. Serious AEs (SAEs) were reported from the time of subject enrollment until study closure. Episodes of *P*. *vivax* relapse were recorded up to the conclusion of the study period, approximately 5 years post-CHMI.

### Immunologic Outcomes

#### Serologic findings

Total immunoglobulin (Ig) G antibodies to VMP001 and other antigens representing different regions of *P*. *vivax* CSP were measured by enzyme-linked immunosorbent assay (ELISA). Immulon 2HB plates (Dynatech, VA) were coated with 0.4 μg/ml of VMP001 or proteins representing the N- and C-terminal regions of *P*. *vivax* CSP, or with 1μg/ml of a synthetic peptide representing the Type 1 repeat region of *P*. *vivax* as described previously [16]. After blocking with casein buffer, plates were incubated with serially diluted serum, followed by horseradish peroxidase (HRP)-labeled goat anti-human IgG (KPL Inc, MD). The reaction was developed using ABTS Peroxidase Substrate System (KPL Inc, MD) and read after 60 min using a SPECTRAmax 340PC Plate Reader (Molecular Devices, CA) at a wavelength of 414 nm. ELISA titers are defined as the serum dilution giving an optical density (OD) of 1.0.

#### Intracellular cytokine analysis

Peripheral blood mononuclear cells (PBMCs) were isolated from blood collected prior to the first immunization, on day 14 after the 2^nd^ and 3^rd^ immunizations, as well as 1 and 6 months post CHMI. PBMCs were separated using Lymphocyte Separation Medium (Lonza, Walkersville, MD) and then cryopreserved in liquid nitrogen until use. After thawing, one million PBMCs were stimulated with antigen in the presence of 1mg/ml anti-CD28 and anti-CD49d for 2 hr at 37°C. GolgiPlug (BD Biosciences, San Jose, CA) was added to the wells, and the incubation resumed for an additional 14 hr at 37°C. Antigens used for stimulation included VMP001 (10μg/ml) and peptides (5μg/ml each of 15-mer peptides overlapping by 11) representing the N-term, repeat, and C-term regions of VMP001. Staphylococcal Enterotoxin B was used as positive control. Cells were washed and stained with anti-CD3-PE, anti-CD4-PerCP, anti-CD8a-V500, anti-CD45RA-Qdot605 (Invitrogen), anti-CD27-V450, and LIVE/DEAD Fixable Dead Cell Stain Kit (Invitrogen). Cells were then washed, permeabilized and stained intracellularly with anti-interleukin (IL)-2-APC, anti-tumor necrosis factor (TNF)-PECy7 and anti-interferon (IFN)-γ- fluorescein isothiocyanante (FITC). Except where indicated, all antibodies were from BD Biosciences, CA. Cells were subsequently washed and acquired on an LSRII FACS machine (BD Biosciences, CA). Data were analyzed using FlowJo software Version 8.8.6 for Macintosh (Tree Star, OR). Time points of major blood draws for humoral and cellular assessments are enumerated in S1 Fig.

### Vaccine Efficacy Outcomes

#### Controlled Human Malaria Infection (CHMI)

In order to evaluate vaccine efficacy, all vaccinated subjects who completed the immunization series underwent CHMI 14 days after the third vaccination. Malaria-naïve control subjects who received no vaccinations underwent CHMI on the same day to ensure challenge model integrity. All subjects were exposed to *P*. *vivax* sporozoites through the bites of 5 infected *An*. *dirus* mosquitoes. Laboratory-reared mosquitoes were membrane-fed on the blood from a single Thai donor who was infected with *P*. *vivax*. Donor blood was screened by PCR to rule out co-infection with other *Plasmodia*. PCR and ELISA assays were used to speciate *P*. *vivax* to ensure that the infection was caused by the type 1 strain of *P*. *vivax*. To prevent inadvertent transmission of blood-borne infections to challenge recipients, donor blood was screened according to FDA (Title 21 of the U.S Code of Federal Regulations (21 CFR) 610.40) and Red Cross (http://www.redcrossblood.org) blood transfusion guidelines. Infected mosquitoes were transported in secure containers from Thailand to the United States and maintained in the insectary at WRAIR until the day of challenge.

Blood smears were performed at least daily beginning on day 5 post-challenge. Volunteers were accommodated in a hotel facilitating access to immediate medical care by trained physicians and nurses starting on day 9 post-challenge. All volunteers were treated when they were diagnosed with malaria by thick blood smear and were monitored in the hotel until they had three negative blood smears on consecutive days.

Subjects with parasitemia were treated for blood-stage infection with a standard course of oral chloroquine (1500 mg base over 48 h) to clear their blood-stage infection. Radical cure of *P*. *vivax* hypnozoites was provided (30 mg primaquine phosphate base by mouth, daily for 14 d) under supervision of study staff. All volunteers cleared their parasitemia as determined by blood smear and PCR following treatment and all completed a 14 day course of primaquine (PQ). Two subjects experienced multiple relapses beginning approximately two months post challenge as previously reported [17]. Subjects experiencing a relapse were re-treated with 1500 mg chloroquine base over 3 days. Radical cure therapy was provided again using PQ by mouth, but to achieve a total dose of 6 mg/kg for volunteers weighing greater than 70 kg [18]. The daily maximum primaquine dose administered was 30 mg by mouth daily.

#### Statistical analyses

All analyses were performed based on a statistical analysis plan (SAP). Statistical tests were 2-tailed where appropriate. Vaccine efficacy (VE) was determined using only those subjects who were challenged. Vaccine efficacy was defined as [(1-R) x 100] where R is a ratio of malaria incidence in vaccinated group relative to the control group. The R was calculated using the Cox proportional hazards model. The median time to parasitemia for each group was considered the prepatent period for the respective group. Kaplan-Meier survival curves were generated for each group and plotted at % uninfected over time. Survival curve comparisons were performed using Log-rank (Mantel-Cox) Test.

## Results

### Study flow

This study was conducted at the WRAIR Clinical Trials Center. A total of 30 subjects received at least one vaccination. Of those, 27 subjects that completed the three dose vaccination regimen, and 6 infectivity control subjects were challenged by the bites of five *P*. *vivax* infected mosquitoes. (Figs 1 and 2).

### Safety Outcomes

**Fig 3:**
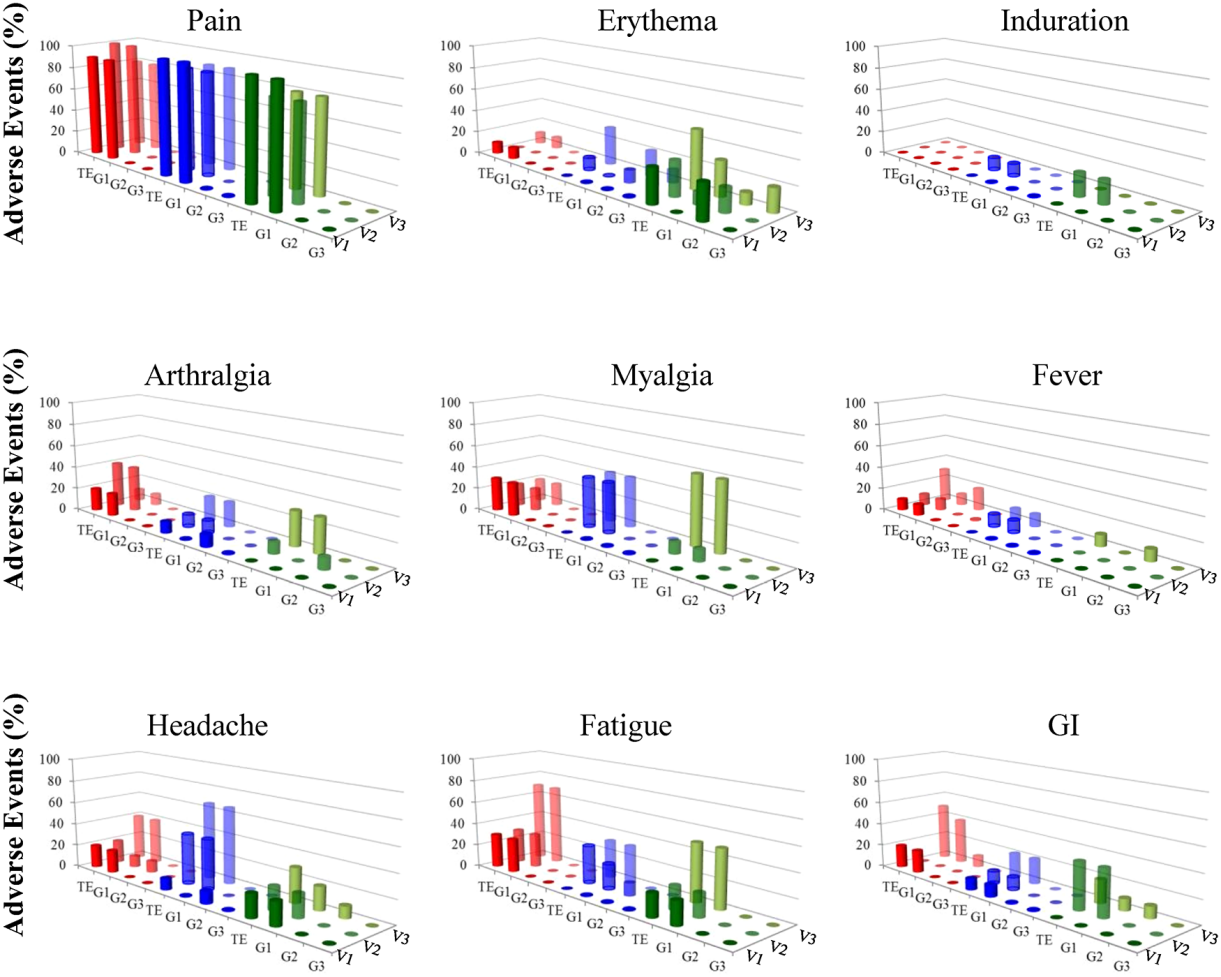
Adverse events. Solicited Adverse Events (AEs) were recorded following each vaccination and are reported as the percentage of subjects in each cohort reporting the event. Pain was the most frequently reported AE, being reported by 80–100% of the subjects. A majority of the AEs presented as Grade 1 (G1, mild). Grade 2 (G2, moderate) erythema, fever, headache and Grade 3 (G3, severe) erythema were seen in a small percentage of individuals. AEs are shown post 1^st^ (V1), 2^nd^ (V2) and 3^rd^ (V3) vaccine dose.

Immunizations of subjects in all three dose cohorts were well tolerated and no safety halting criteria were met. There were no clinically concerning imbalances observed between groups. Similar frequencies of solicited local and solicited general AEs were reported in the three cohorts (Fig 3). There were no AEs in any vaccination cohort that led to withdrawal of any subjects. One SAE (ductal carcinoma in situ of the breast) occurred during the study and it was determined that the event was not related to the study vaccine or CHMI.

Mild (grade 1) to moderate (grade 2) pain in the days following immunization was the most frequently reported local solicited AE, occurring in 100% of subjects who received at least one vaccination. No subjects experienced severe (grade 3) pain. Grade 3 solicited local AEs included erythema at the injection site in 4 subjects, 1 in cohort 2 and 3 in cohort 3. The grade 3 erythema lasted for ≤2266 3 days in 3 of the subjects and in 7 days for one subject. Erythema was not associated with more severe pain or any functional impairment.

Fatigue and headache were the most common solicited systemic AEs following each vaccination, increasing in frequency from vaccination 1 to vaccination 3. The number of subjects experiencing fatigue and headache increased from 17% to 52% and 17% to 45%, respectively, following vaccination 1 to vaccination 3. Myalgia and arthralgia occurred in their highest frequency following vaccination 3, occurring in 41% and 21% of subjects, respectively. Gastrointestinal AEs, to include nausea, diarrhea and abdominal pain, (19%) and fever (10%) were also recorded most frequently following vaccination 3. All other systemic AEs occurred in 10% or less of the subjects following any vaccination. All solicited systemic AEs resolved within the 7-day follow-up period. There was one episode of fever that met the criteria for a grade 3 solicited systemic AE which resolved in less than 24 h. There appeared to be a trend towards increased numbers of solicited AEs associated with each subsequent vaccination. Throughout the vaccination period, eleven mild (grade 1) biochemical or hematologic laboratory adverse events were documented, none of which were determined to be related to vaccination. There were no grade 2 or 3 laboratory abnormalities during this timeframe.

### Challenge Safety

This study represents only the second site to implement a *P*. *vivax* CHMI model which, unlike *P*. *falciparum*, requires mosquitoes that have been fed on blood directly obtained from an infected human donor rather than from in vitro cultured parasites. To ensure subject safety, donor blood was subjected to transfusion-grade screening for blood-borne infections as well as vector-borne infections. All study volunteers were screened to ensure they had normal concentrations of G6PD to prevent hemolytic anemia during radical cure therapy with primaquine. The challenge was well tolerated with no untoward reactogenicity following mosquito bites and 100% of the subjects that were exposed became parasitemic. No untoward SAEs were observed following the challenge and treatment phases.

On days 65 and 79 days post-CHMI, 2 subjects experienced *P*. *vivax* relapse [17] which we hypothesize was associated with their inability to metabolize PQ into sufficiently high enough concentrations of its active form leading to drug failure. One subject experienced a total of two relapses while the second subject experienced three relapses. No further relapses were observed up to the end of the study period, approximately 5 years post CHMI.

### Immunogenicity Outcomes

Seroconversion to VMP001 following the second vaccination was set as the progression criteria for proceeding to CHMI. Subjects were considered to have seroconverted if, at a serum dilution of 1:100, the OD_414_ of the test sample obtained two weeks post-2^nd^ immunization was significantly different by paired t-test compared to serum obtained prior to 1^st^ immunization (data not shown). All subjects seroconverted, with anti-VMP001 antibodies detectable in 100% of vaccinees at 2 weeks post vaccine dose 2.

**Fig 4:**
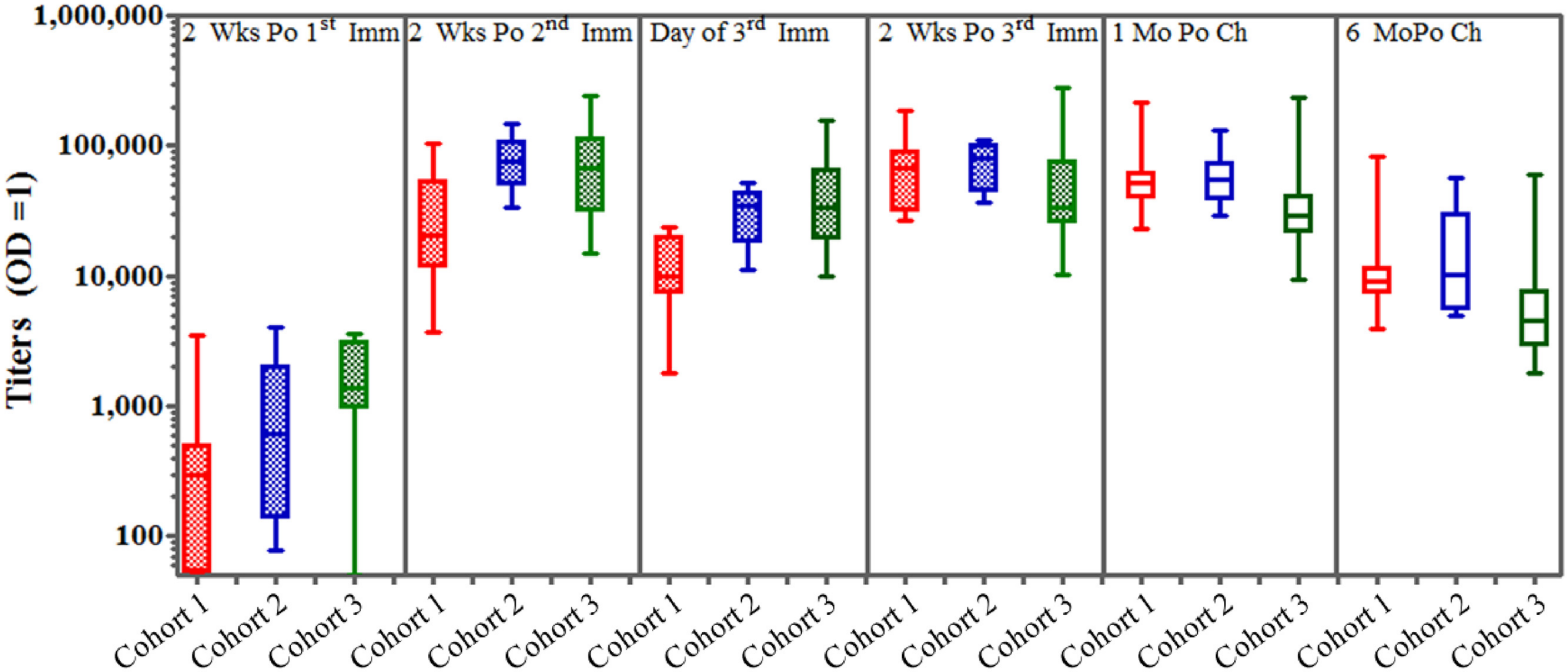
All volunteers immunized with VMP001/AS01_B_ generated antibodies to VMP001. Anti-VMP001 antibodies were detected in 80% of vaccinated individuals starting at two weeks post-1^st^ immunization. Titers were boosted to peak levels post-2^nd^ immunization, with 100% of subjects developing antibodies. A decrease in antibody titers was observed on the day of third immunization (8, 6 and 4 weeks post 2^nd^ immunization for cohorts 1, 2 and 3, respectively) followed by a slight increase post 3^rd^ immunization. Antibody titers, defined as a serum dilution that gives an OD_414_ of 1.0, showed a continual decline post challenge (Po Ch). Box plot represents the 25–75 percentiles and Whiskers indicate the minimum and maximum values. GMTs of anti-VMP001 antibody were significantly higher in group 1 compared to group 2 at 2 time points (2weeks post 2nd, p = 0.01, and on the day of 3rd, vaccination, p = 0.002). GMTs of anti-VMP001 antibody were significantly higher in group 2 compared to group 3 at 2 time points (4weeks post DOC, p = 0.03, and 6 months post-DOC, P = 0.04). GMTs of anti-VMP001 antibody were significantly higher in group 1 compared to group 3 at 5 time points (2 weeks post 1st vaccination, p = 0.02, on the day of 2nd vaccination, p = 0.01, 2 weeks post 2nd vaccination, p = 0.04, on the day of 3rd vaccination p = 0.002, and 6 months post DOC, p = 0.034).

Having met the progression criteria, subsequent ELISA data was reported as antibody titer, defined as the reciprocal of the serum dilution giving an OD_414_ of 1.0, and responses were measured at 2 weeks following each immunization as well as at 1 and 6 months post challenge. The highest geometric mean titer (GMT) of anti-VMP001 antibody were noted 2 weeks after dose 2 in cohorts 2 and 3 (74,608 and 61,711, respectively) and on the day of challenge (DOC; 2 weeks after dose 3) in cohort 1 (61,203) (Fig 4). Peak GMTs of anti-VMP001 antibody were not significantly different between the groups. While there was a decrease in antibody titers in all three cohorts in the weeks following the 2^nd^ immunization, titers were boosted minimally (for cohort 3) to significantly (for cohort 1), following the 3^rd^ immunization. As a result, GMTs were not statistically significantly different between cohorts 1, 2, and 3 on the DOC (Fig 4). Antibody titers showed a 5 to 8-fold decrease 6 months post-challenge compared to the pre-challenge titers. However, these titers remained significantly higher than those measured following the 1^st^ immunization.

### Antibody fine-specificity

**Fig 5:**
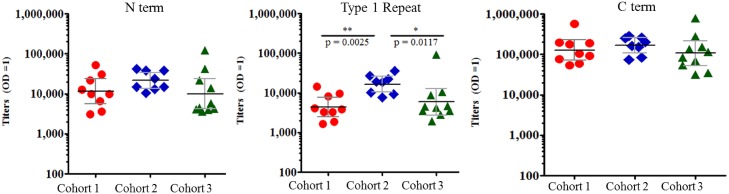
Fine-specificity analysis demonstrates that all domains of CSP are recognized following immunization with VMP001. Day of Challenge (DOC, or 2 weeks post-3^rd^ vaccination) sera from all subjects were reactive to the N-term, type-1 Repeat peptide, as well as C-term region of the protein with the highest reactivity to the C-term. C- term GMTs were significantly higher in all three cohorts compared to the N- term and Type 1 repeat GMTs. Cohort 2 had significantly higher anti-Repeat antibodies compared to Cohorts 1 and 3 (p = 0.003, p = 0.01, respectively).

Antibody fine-specificities were evaluated to determine if the antibody responses were skewed to any specific region of the protein. Antibodies were detected to all regions of the protein, i.e. to the N-terminal, central repeat region as well as the C-terminal region. There were no significant differences in the GMT of anti-C term or anti-N term antibody between any groups on the DOC. The DOC GMT of anti-Type 1 repeat antibody was significantly higher in group 2 (GMT 16,554) compared to groups 1 and 3 (GMT 4,412 and 5,922 respectively) (Fig 5). There were no significant differences in GMT of anti-Type 1 repeat antibody between groups 1 and 3 on the DOC.

### Cell mediated immune (CMI) response to VMP001

**Fig 6:**
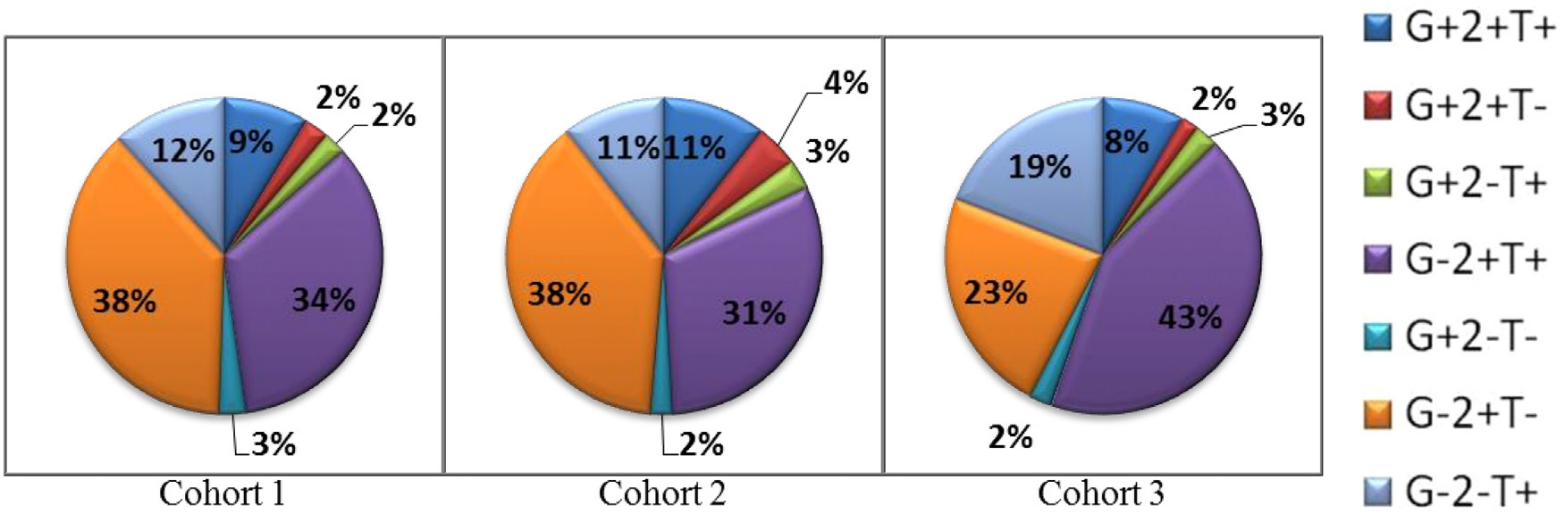
Intracellular cytokine analysis shows the presence of antigen-specific cytokine production by CD4+ cells. Peripheral blood mononuclear cells from vaccinated subjects were stimulated with VMP001 and production of IL-2, IFN-γ and TNF-α was assessed. Pie chart depicts the percentage of CD4^+^ cytokine-producing cells for each cohort 2 weeks post 3^rd^ vaccination (Day of challenge). G, 2 and T indicate IFN-γIL-2 and TNF-α, respectively. Cytokine producers are represented by a “+” and non-producers by a “-”.

CD4+ T cell responses to VMP001 were detected in all individuals, with a majority (60%) showing peak response 14 days after the 2^nd^ immunization. All vaccinated individuals produced IL-2, 93% produced TNF-α and 55% produced IFN-γ following stimulation with VMP001. Cytokine positive cells were predominantly IL2+ single positive or IL2+TNF-α+ double positive (Fig 6). Smaller frequencies of triple positive cells that also expressed IFN-γ were also detected (Fig 6). Cytokine profiles did not show marked differences post challenge. The responses were predominantly directed towards the N-term region (90% volunteers). Only 17% of vaccinated subjects responded to the repeat and C-term regions. There were no detectable CD8+ T cell responses in any volunteer at any of the time-points tested.

### Vaccine efficacy

**Fig 7:**
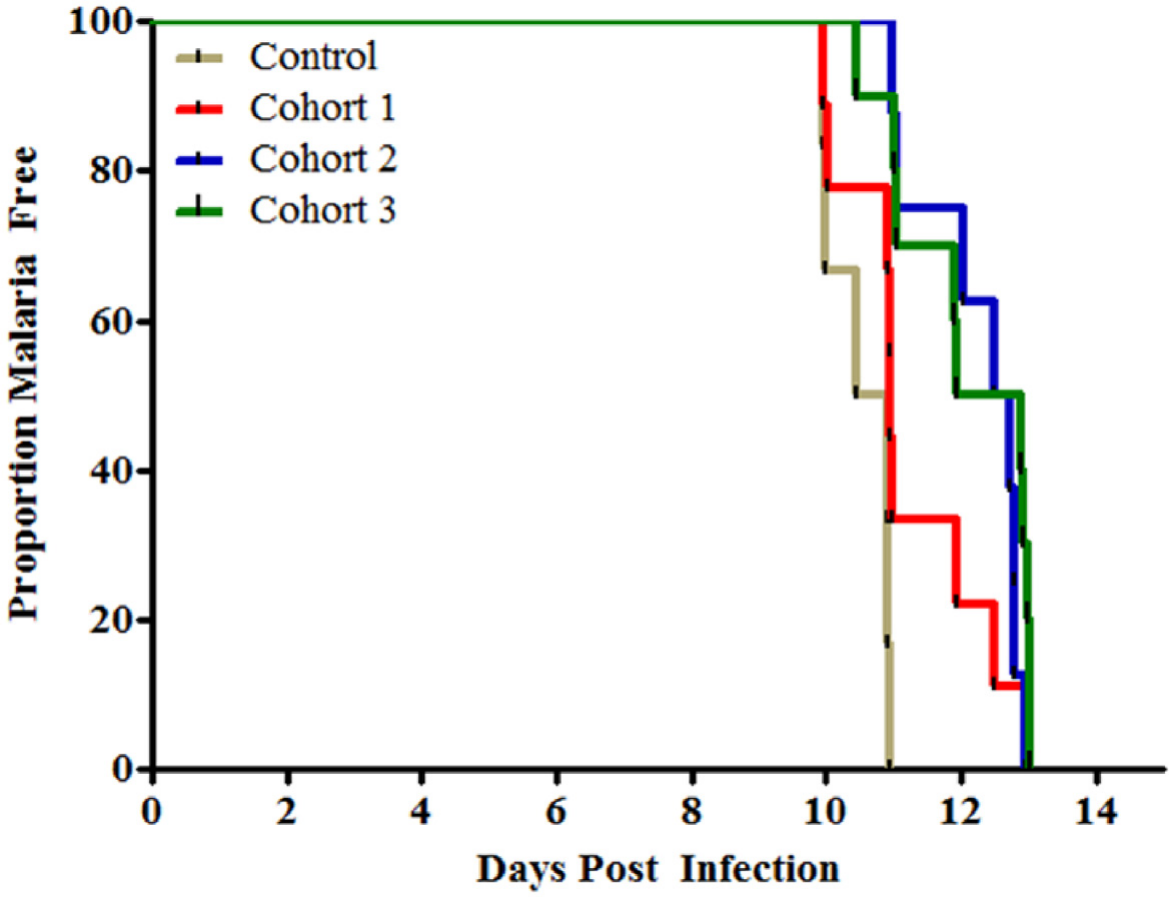
Kaplan-Meier survival curves were used to plot time to parasitemia. Nine volunteers from cohort 1 (red), 8 volunteers from cohort 2 (blue) and 10 volunteers from cohort 3 (green) were challenged along with 6 non-vaccinated controls via the bites of 5 of Anopheles dirus mosquitoes infected with P. vivax parasites originating in Thailand. The median time to parasitemia for all three vaccinated cohorts was significantly longer compared to the infectivity controls with cohort 2 having the longest delay in median time to patency (12.6 days).

Following CHMI, all infectivity control subjects (100%) became infected. All 27 (100%) immunized subjects who underwent CHMI also became infected (Fig 7). Vaccine efficacy was 0%. The median prepatent period of all immunized subjects was 11.9 days and that of infectivity controls was 10.7 days. A significant delay in the median prepatency period was noted in all cohorts as compared to the infectivity controls (cohort 1, 10.9 days, p = 0.0261; cohort 2, 12.6 days, p<0.0001; cohort 3, 12.4 days, p = 0.0003). The median prepatency period for cohort 3 was significantly longer than that of cohort 1 (p = 0.035; Log-rank test); otherwise, there was no statistically significant delay in median prepatency periods between the other vaccinated groups.

**Fig 8:**
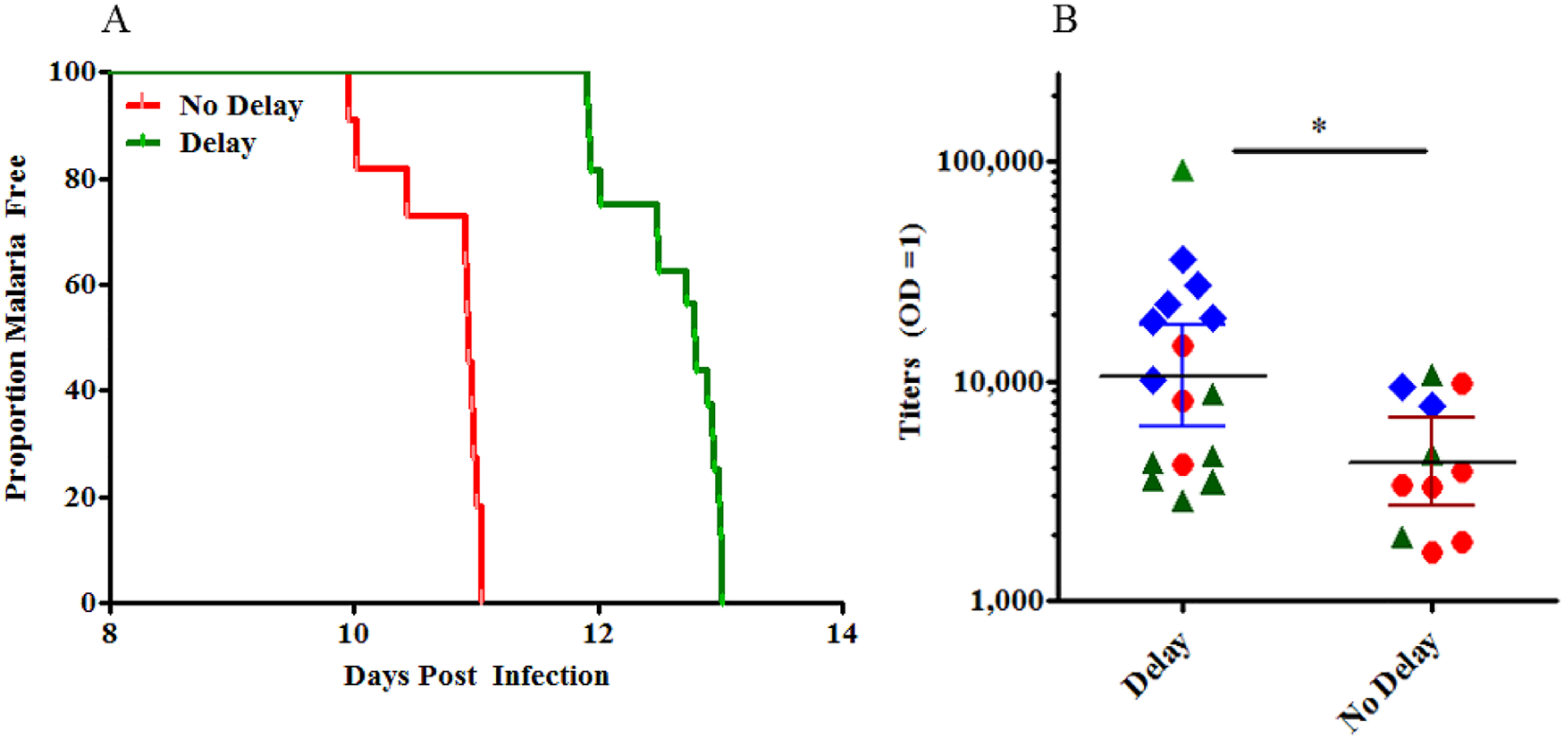
Vaccinated subjects who showed a delayed time to parasitemia had higher anti-Type 1 repeat antibodies. A. Vaccinated individuals from all three cohorts could be grouped into those that showed a delay (green, n = 16) compared to the controls, and those that did not show a delay (red, n = 11). B. Individuals with a delay in patency had higher anti-type 1 repeat antibodies compared to those with no delay (p = 0.02). Each symbol represents an individual. Cohorts 1, 2 and 3 are represented by red, blue, and green, respectively.

Vaccinated subjects with a prepatency period greater than 2 standard deviations above the mean of the prepatency period of the infectivity controls were categorized as having a significant delay in the onset of parasitemia. The median time to parasitemia for the delayed group (n = 16) was 12.8 days and 10.9 days in the subjects without delay (n = 11) (Fig 8A).

On the day of the CHMI there was a significant correlation (r = 0.51, p = 0.006) in all vaccinated subjects between anti-type 1 peptide antibody titer and net time to parasitemia (time to parasitemia of vaccinated subjects—time to parasitemia of control subjects). The GMT of anti-type 1 repeat antibody was significantly greater in the delayed group (10,489, 95% CI = 6,154–17,878) as compared to the group who did not experience delay (4,279, 95% CI = 2,684–6,822) (p = 0.025, 2-tailed Mann Whitney t-test) (Fig 8B). There were no other significant differences in the GMT of anti-VMP001, anti-N term, or anti-C term antibody between the delayed and no delay groups.

No significant differences were identified between the cohorts in terms of CMI response at any of the time points and there was no association between CMI and delay in prepatency period.

## Discussion

The development of a controlled human malaria infection challenge model to evaluate *P*. *vivax* vaccines is challenging and fraught with numerous technical obstacles. While *P*. *falciparum* has been adapted for use in CHMI primarily because of the ability to grow mature gametocytes that infect *Anopheles* mosquitoes to produce infectious stage sporozoites, the same methodology cannot be used for *P*. *vivax*. To date, *P*. *vivax* has proven refractory to continuous in vitro culture. Therefore, infection of permissive Anopheline mosquito species (i.e. *An*. *dirus* and *An*. *albimanus*)—that themselves are difficult to maintain and/or infect in insectaries—relies on the identification and consent of naturally-infected patients to present to medical treatment facilities to donate blood for initiating infection in mosquitoes. Nevertheless, the successful implementation of *P*. *vivax* CHMI previously reported in Colombia has opened the door to test vaccine efficacy by incorporating an infected mosquito challenge [19]. In preparation for conducting a vaccine efficacy study we established a CHMI model for *P*. *vivax* at WRAIR, in collaboration with AFRIMS, using mosquitoes that were infected in Thailand (Chuang et al. in preparation.

Here we report the initial safety, immunogenicity, and efficacy data for the candidate vaccine VMP001/AS01_B_. This represents the first human efficacy study incorporating a CHMI for any *P*. *vivax* vaccine.

This vaccine formulation, administered in 3 increasing antigen doses (15 μg, 30 μg, or 60 μg) was well tolerated and the adverse event profile was consistent with other vaccines containing the AS01_B_ adjuvant system [10,20,21].

The vaccine induced strong antibody and CD4+ T cell immune responses in all antigen dose groups. While all groups demonstrated greater than 50-fold boosting in antibody titers between the first and second immunization, only the low dose cohort showed a modest 2.8-fold increase in titers between the second and third immunization, thus matching the titers of the other two cohorts, which did not show any boost in antibody titers post third immunization. A possible explanation for the absence of antibody boosting following the second vaccination in the medium- and high-dose cohorts could be that the antibody titers were already saturated for these cohorts and either the vaccine dose or the range of intervals between the 2^nd^ and 3^rd^ vaccination (6 and 4 weeks, respectively) did not allow for sufficient enhancement in titers.

No subjects were sterilely protected following CHMI; however, all dose groups experienced a statistically significant delay in mean prepatency period as compared to the infectivity control group suggesting an anti-parasite effect elicited by the vaccine. A two day delay in prepatent period reflects a significant decrease in liver-stage parasites [22] [23], indicating that the immune responses generated by the vaccine is able to induce partial protection in vaccinated subjects. This would be consistent with the previously described correlation of high *P*. *falciparum* CSP repeat-specific antibody titer with sterile protection in malaria naïve adults [10] and children in endemic regions [11,12]. We have previously reported an efficacy study in *Aotus* monkeys that were immunized with VMP001 formulated in Montanide ISA 720 and CpG. Following an intravenous challenge a vaccine efficacy of 66.7% was observed and this protection was associated with anti-type 1 antibodies [24]. This observation supports the results observed in the current study. The lack of protection in humans may be due to a lower magnitude of anti-type 1 antibody titers in comparison to those observed in the *Aotus* study which was conducted with a different adjuvant formulation (Yadava, A. manuscript in preparation).

As we consider strategies to improve vaccine efficacy, alternate approaches, such as particulate delivery to improve immunogenicity and epitope-display, and well as alterations in schedule and dosing to improve qualitative and quantitative responses are points to ponder. We have developed CSV-S,S, a particulate formulation containing VMP001 which, similar to the *P*. *falciparum* CSP-based vaccine RTS,S, is co-expressed as a hepatitis B fusion particle. Analysis of the fine specificity of antibody responses in serum from rhesus monkeys immunized with CSV-S,S demonstrated significantly higher antibody response to the type 1 repeat peptide as well as greater responses to a smaller AGDR sequence within the type 1 peptide, suggesting that a particle formulation may improve the humoral response to the repeat sequences over soluble protein alone in the presence of adjuvants that are appropriate for human use [16].

The significant association of type 1 repeat-specific antibody responses with delay in prepatency period suggests that new vaccine strategies that enhance immune responses to this region might further improve vaccine efficacy against these strains of *P*. *vivax*. In addition to the modulation of immune responses to the repeat region by particulate formulations, an alternate strategy is to increase the number of repeat motifs in the vaccine construct. The resulting increase in epitope density may result in enhanced anti- repeat-specific responses.

Finally, alterations in schedule and dosing to optimize antibody affinity to protective epitopes may improve vaccine efficacy as has been reported for the RTS,S vaccine (Regules et al. in preparation).

The logistical difficulties of performing *P*. *vivax* CHMI have slowed the developmental efforts for a vaccine against this parasite. We demonstrate that this challenge model, although complex, is feasible and can provide rapid assessment of vaccine efficacy. An unexpected outcome from the CHMI in this study identified two subjects who experienced multiple relapses from latent hypnozoites parasites despite adherence the optimal radical cure therapy. The investigation into the cause and follow-up of these two subjects has been reported previously and identified an association between the cytochrome P450 isoenzyme 2D6 (CYP2D6) phenotype and the metabolism of PQ. It appears that CYP2D6 poor metabolizers are unable to convert the parent drug PQ into its active metabolite responsible for anti-hypnozoite activity and are, therefore, more likely to experience PQ failure and *P*. *vivax* relapse [13]. We propose that in addition to the Duffy blood group and G6PD testing, a laboratory screening test be used to characterize volunteers’ CYP2D6 genotype/phenotype in order to exclude subjects who, based on their genetic background, would be more likely to fail PQ therapy and experience relapse.

Decreases in *P*. *falciparum* malaria especially in Southeast Asia have not been associated with commensurate decreases in *P*. *vivax* malaria. Recent strategies to eliminate *P*. *vivax* by targeting the reservoir of latently infected patients with antimalarial 8-aminoquinolines alone are unlikely to achieve elimination because of both the safety and lack of active drug metabolites in a significant proportion of the population [13]. A highly protective and durable pre-erythrocytic CSP-based *P*. *vivax* vaccine would have a dual beneficial effect of preventing not only the initial infection but also secondary relapses from hyponozoites thus inhibiting the establishment of latent infection.

## Supporting Information

S1 Fig Immunological assessments. Flow diagram indicating time points of the major blood draws during the course of the study. Blood samples were collected for humoral (serum) and cellular (peripheral blood mononuclear cells) analysis at two weeks post (Po) each immunization for each cohort as well as one and 6 months post challenge.
(PDF)
