# Supplementary figures and images for: Phase 1/2a Trial of Plasmodium vivax Malaria Vaccine Candidate VMP001/AS01B in Malaria-Naive Adults: Safety, Immunogenicity, and Efficacy
Source: PLoS Negl Trop Dis. 2016 Feb 26;10(2):e0004423. doi: 10.1371/journal.pntd.0004423 (PMC4769081; doi:10.1371/journal.pntd.0004423)

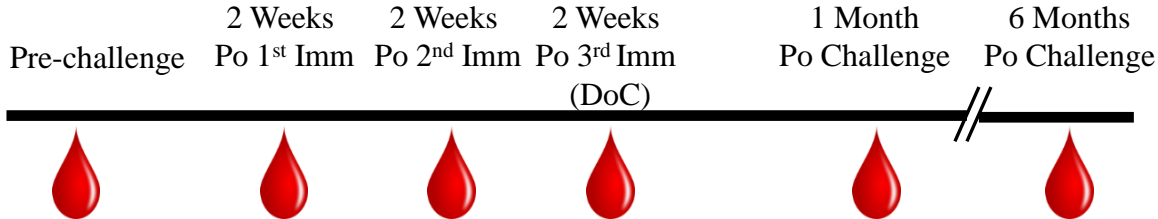

Supplement: S1 Fig — Flow diagram indicating time points of the major blood draws during the course of the study. Blood samples were collected for humoral (serum) and cellular (peripheral blood mononuclear cells) analysis at two weeks post (Po) each immunization for each cohort as well as one and 6 months post challenge. (PDF) [file pntd.0004423.s001.pdf]
